# A Possible Mechanism of Metformin in Improving Insulin Resistance in Diabetic Rat Models

**DOI:** 10.1155/2019/3248527

**Published:** 2019-10-13

**Authors:** Mengsiyu Li, Xiaowen Hu, Yeqiu Xu, Xiaolin Hu, Chunxue Zhang, Shuguang Pang

**Affiliations:** ^1^School of Medicine and Life Sciences, University of Jinan-Shandong Academy of Medical Sciences, Jinan, China; ^2^Department of Infectious Diseases, Jinan Central Hospital Affiliated to Shandong University, Jinan, China; ^3^Department of Endocrinology, Jinan Central Hospital Affiliated to Shandong University, Jinan, China; ^4^Department of Radiology, Jinan Central Hospital Affiliated to Shandong University, Jinan, China

## Abstract

**Background:**

Type 2 diabetes has become one of the most common diseases worldwide, causing a serious social burden. As a first-line treatment for diabetes, metformin can effectively improve insulin resistance. It has been reported that 12*α*-hydroxylated BA (mainly CA) is associated with insulin resistance. The purpose of this study was to analyze the changes in CA and possible signaling mechanisms in diabetic rats after metformin intervention.

**Methods:**

HepG2 cells were cultured after adding different concentrations of metformin. The cell viability was measured using CCK8 kit, and the expression of FXR, MAFG, and CYP8B1 in cells was detected by WB. The rat models of type 2 diabetes were induced by low-dose streptozotocin by feeding a high-fat diet, and the control rats (CON) were fed on normal food; the diabetic rats (DM) were given a high-fat diet without supplementation with metformin, while the metformin-treated diabetic rats (DM + MET) were given a high-fat diet and supplemented with metformin. Biochemical parameters were detected at the end of the test. Expression levels of FXR, CYP8B1, and MAFG were assessed by WB. Serum CA were measured using an enzyme-linked immunosorbent assay (ELISA).

**Results:**

In HepG2 cells, metformin dose-dependently enhanced the transcriptional activity of FXR and MAFG and inhibited the expression of CYP8B1. Metformin-treated DM rats showed improved glucose and bile acid metabolism. In addition, significantly increased FXR and MAFG and decreased CYP8B1 were observed in DM + MET rats. At the same time, the CA content of metformin-treated rats was lower than that of diabetic rats.

**Conclusion:**

Changes in CA synthesis after metformin treatment may be associated with inhibition of CYP8B1. These results may play an important role in improving insulin sensitivity after metformin treatment.

## 1. Introduction

T2DM is a common complex metabolic disorder. Insulin resistance and relatively insufficient insulin secretion are major features of type 2 diabetes. As a first-line antidiabetic drug, metformin mainly reduces hyperglycemia and improves glucose uptake and insulin sensitivity by inhibiting gluconeogenesis. Studies have shown that insulin resistance may be associated with 12*α*-hydroxylated bile acids [1]. The compositional ratios and related principles of 12*α*-hydroxylated BA after metformin treatment may play a major role in alleviating insulin resistance.

Sterol 12*α*-hydroxylase (CYP8B1) is an essential enzyme that promotes the synthesis of 12*α*-hydroxylated bile acids [2]. Unlike CYP7A1, which is the rate-limiting enzyme of the classical pathway of bile acid metabolism, CYP8B1 is mainly responsible for the synthesis of 12*α*-hydroxylated bile acids and controls the ratio of cholic acid in bile to that of chenodeoxycholic acid, among which cholic acid is the most abundant 12*α*-hydroxylated bile acid in liver tissue [3]. Farnesol X receptor (FXR) is an activated transcriptional regulator of bile acid and glucose metabolism [4]. In addition, FXR-mediated SHP inhibits CYP7A1 expression [5]. In contrast to the detailed study of the mechanism by which metformin inhibits CYP7A1 [6, 7], the mechanism by which metformin inhibits CYP8B1 and cholic acid synthesis remains unclear. Recent studies have confirmed that MAFG is a target of FXR and a key transcriptional repressor of bile acid synthesis and metabolism [8–11]. MAFG has been shown to reduce the transcription level of CYP8B1 in the liver [12].

Bile acid profile is a key regulator of metabolic pathways. Metformin inhibits the expression of the enzyme (CYP8B1) required for the synthesis of 12*α*-hydroxylated bile acid and inhibits the synthesis of cholic acid, which may be effective targets for the treatment of type 2 diabetes [13].

## 2. Materials and Methods

### 2.1. Reagents and Antibodies

DMEM medium (HyClone Corp.), fetal bovine serum (Sijiqing), metformin hydrochloride (Sigma, USA), CCK8 kit (Sigma, USA), FXR antibody (Biorbyt), CYP8B1 antibody (Abcam), CYP7A1 antibody (Abcam), and MAFG antibody (Abcam) were used in the experiments.

Metformin from Bristol-Myers Squibb and STZ from Sigma (USA), as well as metformin and compound C from Sigma-Aldrich Co. (St Louis, MO, USA) were employed in the experiments.

### 2.2. Cell Culture

Human hepatoma HepG2 cells were acquired from the Cell Bank of Shandong Academy of Medical Sciences (Shandong, China). HepG2 cells were cultured in high glucose-DMEM containing 10% fetal bovine serum and 1% penicillin/streptomycin. The cells were incubated in a moist atmosphere of 5% CO_2_ at 37°C and passaged every 3 days by trypsinization.

### 2.3. Cell Proliferation Assay

The cytotoxic effect of metformin was evaluated using a CCK8 assay. HepG2 cells were sowed into 96-well plates at a density of 1 × 10^4^ per well and were incubated at 37°C for 24 h in a humidified atmosphere of 5% CO_2_. Then different concentrations (0, 0.5, 1, 1.5, and 2 mM) of metformin were added. After 24 h of culture, a cell proliferation assay was performed using Cell Counting Kit-8 (CCK-8) (Sigma, USA) according to the manufacturer's illustrations. The absorbance was read at 450 nm with a microplate reader (Bio-Rad).

### 2.4. Cultured Cells

In order to prove the influences of metformin intervention on FXR, MAFG, and CYP8B1 expression, metformin was added to the medium in six-well plates containing HepG2 (5 × 10^5^ cells/ml) with the following ultimate concentrations: 0, 0.5, 1, 1.5, and 2 mM for 24 h. In addition, cells were cultured with 1 mM metformin for 0, 12, 24, or 48 h. The cells were harvested for western blotting.

### 2.5. Animal Experiments

Male Wistar rats, weighing 200–230 g, were obtained from Vital River Laboratory Animal Technology Co.Ltd. (Beijing, China), at 8 weeks of age and housed at 23 ± 2°C with a 12-h cycle of light/dark. Water and food were given ad libitum. All animal methods in the experiment were approved by the Animal Research Committee of Shandong University. After one week adaptation period, sixty Wistar rats were randomly distributed into three groups: control rats (CON) group (*n* = 20), diabetic rats (DM) group (*n* = 20), and metformin-treated diabetic rats (DM + MET) group (*n* = 20). For the CON group, rats were fed with a standard diet (6% fat, 64% carbohydrate, and 23% protein). For the DM and DM + MET groups, rats were fed with a HFD (25% fat, 48% carbohydrate, and 20% protein). After HFD feeding for 10 weeks, DM and DM + MET rats were injected intraperitoneally 35 mg/kg body weight streptozotocin (STZ) dissolved in citrate buffer (pH 4.2) to induce a type 2 diabetes model. CON rats were given an equal volume of saline intraperitoneally. Seventy-two hours after STZ injection, the fasting blood glucose (FBG) level was measured by a glucometer. The FBG ≧11.1 mmol/L was regarded as successful induction of diabetes and selected for further studies.

DM + MET rats were administered metformin (500 mg·kg^−1^·d^−1^) intragastrically for 12 weeks after diabetes model induction; CON rats and DM rats were given an equal volume of saline intraperitoneally. FBG levels of the three groups of rats were recorded every morning, and the mental state, food intake, water intake, and body mass were measured. At 32 weeks, all rats were fasted one night before serum and tissue collection. The serum samples and tissues obtained were stored at −80°C.

### 2.6. Food and Water Intake Measured

5 rats were housed in each cage, and 200 g of feed was weighed and placed in a cage. At the same time on the second day, the remaining feed was taken out on the tray and the remaining value was read. The amount of feed added minus the remaining amount is the total food intake of the rat. The total food intake of the rats was divided by the number of rats, ie., the average food intake per rat in the cage.

Two drinking bottles were placed in each cage , each containing 150 ml of water. At the same time on the next day, the water was collected into a graduated cylinder and the remaining water was read. The amount of water added minus the remaining amount is the total amount of water in the caged rats. The total amount of water in the cage was divided by the number of rats, which is the average water intake per rat in the cage.

### 2.7. Oral Glucose Tolerance Test (OGTT), Glucose Concentration, and Insulin Concentration

At 24 weeks, an OGTT was performed after an overnight fast (12 h). A solution of 50% glucose (2.5 g/kg) was administered orally, and four blood samples were obtained from the retrobulbar venous plexus at 0, 30, 60, and 120 minutes for the measurement of glucose and insulin, respectively. Glucose concentration was evaluated using a glucometer (Roche, Basel, Switzerland). Insulin concentration was measured with an ELISA kit purchased from Mercodia (Uppsala, Sweden).

### 2.8. Biochemical Analysis

At the end of the test, the rats were fasted for 16 hours. Blood samples from the eye angular vein were analyzed to evaluate the levels of parameters of total bile acids (TBAs), low-density lipoprotein cholesterol (LDL-C), total cholesterol (TC), high-density lipoprotein cholesterol (HDL-C), and triglyceride (TG). They were tested by cobas 8000 automatic biochemistry analyzer (Roche, Basel, Switzerland). Glucose level was tested by using a glucometer. Insulin was detected with ELISA kits. The steady-state model assessment of insulin resistance (HOMA-IR) index was calculated as [FBG (mmol/L) × FIN (*μ*U/mL)]/22.5.

### 2.9. Western Blotting

Homogenates of rat liver or HepG2 cell lysates were prepared for western blot analysis. Samples were centrifuged at 10,000 rpm for 10 min at 4°C, and the supernatant was collected. Total protein concentration was measured by BCA. The proteins were loaded equally on 12% SDS-PAGE and transferred onto PVDF membranes (0.2 mm, Millipore, Billerica, MA, USA). Membranes were blocked in PBST/5% nonfat dry milk powder and were incubated overnight at 4°C with the primary antibodies against FXR, MAFG, CYP8B1, CYP7A1, and *β*-actin. The secondary antibodies binded to HRP were then incubated with the membranes at room temperature for 45 min. After removal of the secondary antibodies, the blots were washed by an ECL detection kit (Millipore, Billerica, MA, USA) and imaged on an automated gel imaging analysis system. Image J (National Institutes of Health, Bethesda, MD, USA) was used for quantification of immunoblots.

### 2.10. Detection of Cholic Acid in Liver Tissue by ELISA

1 g of liver tissue sample was weighed, 9 g of PBS with a pH of 7.2–7.4 was added, and the sample was homogenized by hand or a homogenizer. Centrifuge for approximately 20 min (2000–3000 r/min), and collect the supernatant carefully. A portion was packed for testing and the rest was frozen for later use. If a precipitate forms during storage, it should be centrifuged again. Cholic acid was detected using an ELISA kit.

### 2.11. Statistical Analysis

Data were calculated from at least three independent experiments and were expressed as mean ± SEM. Comparisons among three groups were obtained via one-way analysis of variance (ANOVA) followed by Dunnett's test. *p* < 0.05 was considered statistically significant. GraphPad Prism .6.0 (GraphPad Software Inc., San Diego, CA) was used to perform the data analysis.

## 3. Results

### 3.1. Effect of Various Doses of Metformin (Met) on Cell Viability

**Figure 1:**
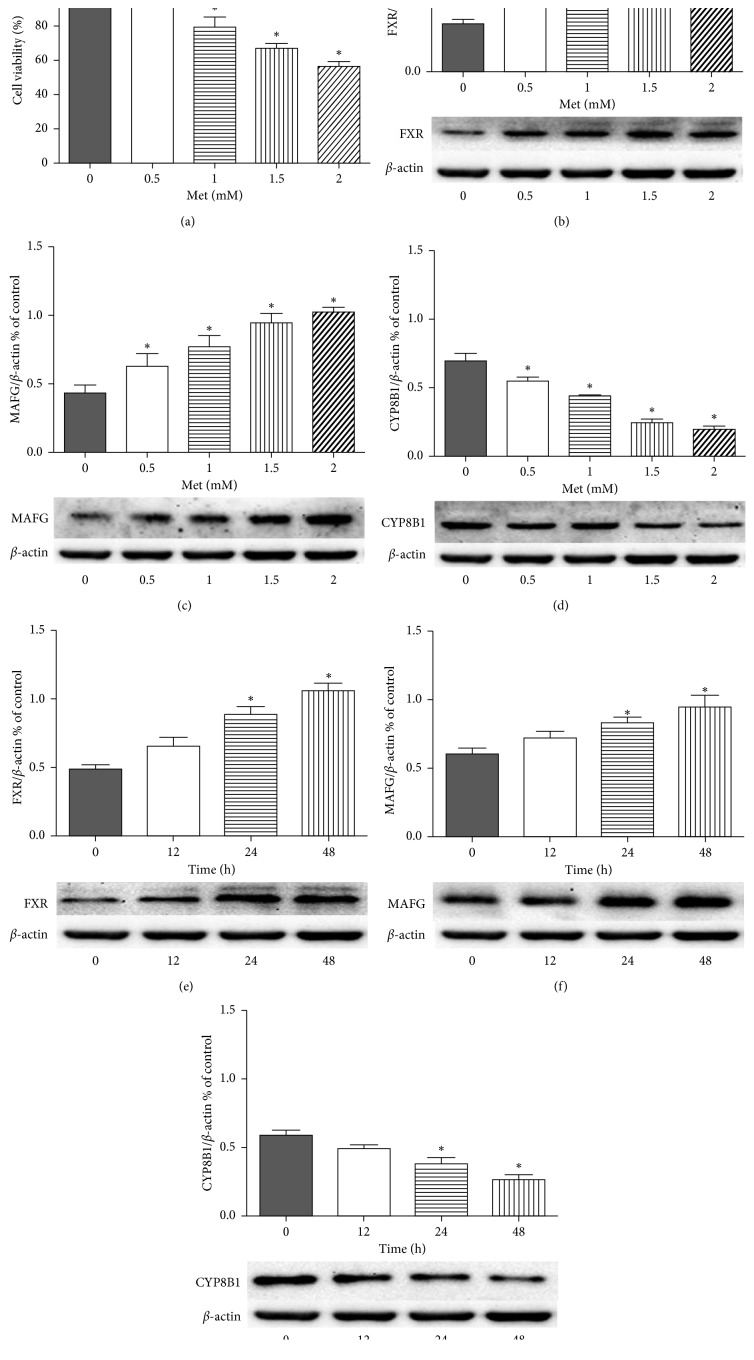
Cell viability and protein expression of FXR, MAFG, and CYP8B1 in each group by metformin treatment in HepG2 cells. (a) The effect of different doses of metformin (Met) on cell viability. Cells were treated with metformin for 96 hours, and the cell viability was measured by CCK8 assay. Results are mean ± SEM; ^*∗*^*p* < 0.05 vs. control group. Protein contents were detected by western blotting and were quantified with Image J *β*-actin was used as a reference to verify equal loading of protein sample. The HepG2 cells were incubated with different concentrations of metformin (0, 0.5, 1, 1.5, and 2 mM) for 24 h quantification of FXR (b), MAFG (c), and CYP8B1 (d). The HepG2 cells were incubated with 1 mM metformin for various durations (0, 12, 24, or 48 h), quantification of FXR (e), MAFG (f), and CYP8B1 (g). Results are mean ± SEM; ^*∗*^*p* < 0.05 vs. control group.

In order to assess the effect of metformin on HepG2 cells, we treated HepG2 cells with various concentrations of metformin for up to 96 hours. We then determined cell growth using the CCK8 assay. As the dose of metformin increased, we observed a significant decrease in the activity of HepG2 cells (Figure 1(a)).

### 3.2. Effects of Various Concentrations of Metformin on FXR, MAFG, and CYP8B1 at HepG2 Cell Level

In order to study the effects of metformin on FXR, MAFG, and CYP8B1 at the cell level, the cells were incubated with metformin at different concentrations (0, 0.5, 1, 1.5, and 2 mM) for 24 hours or with 1.5 mM metformin for various durations (0, 12, 24, or 48 h). As shown in Figure 1, the protein expression levels of FXR, MAFG, and CYP8B1 were measured after incubation with various metformin concentrations for different durations. Western blot analysis showed that metformin promoted the expression of FXR (Figures 1(b) and 1(e)) and MAFG (Figures 1(c) and 1(f)) in a time- and dose-dependent manner, while inhibiting the protein expression level of CYP8B1 (Figures 1(d) and 1(g)). These results suggest that metformin intervention can enhance the sensitivity of FXR and the transcriptional activity of MAFG in a dose-dependent manner and inhibit the expression of CYP8B1 in HepG2.

### 3.3. Effect of Metformin on Food and Fluid Intake and Body Weight of Diabetic Rats

We divided the experiment into two parts: before and after the formation of the diabetes model; the food and fluid intake and body weight of each group of rats were recorded every day (Table 1). During the experiment, HFD/STZ induced type 2 diabetes (DM), the rats were inferior, the body was thin, and the coat was not shiny. It is expressed as a diet and the amount of drinking water is significantly increased, which is consistent with the characteristics of type 2 diabetes. After treatment with metformin, the diabetic rats improved their spirits, and the amount of water and food intake decreased compared with the DM group, but still more than the normal control group.

The fluid and food intake as well as body weight of high-fat diet DM and DM + MET rats were significantly higher than those of the control rats before modeling. There was no significant difference in fluid and food intake and body weight between the DM rats and the DM + MET rats. After successful modeling, DM rats showed weight loss compared to control rats, and DM + MET rats lost weight compared to DM rats after treatment with metformin. Our results indicate that metformin can reduce the body weight of diabetic rats. Inhibition of appetite and reduction of food intake in diabetic rats are its main mechanism.

### 3.4. Effects of Metformin on FBG, Insulin, and Biochemical Parameters of Diabetic Rats

**Figure 2:**
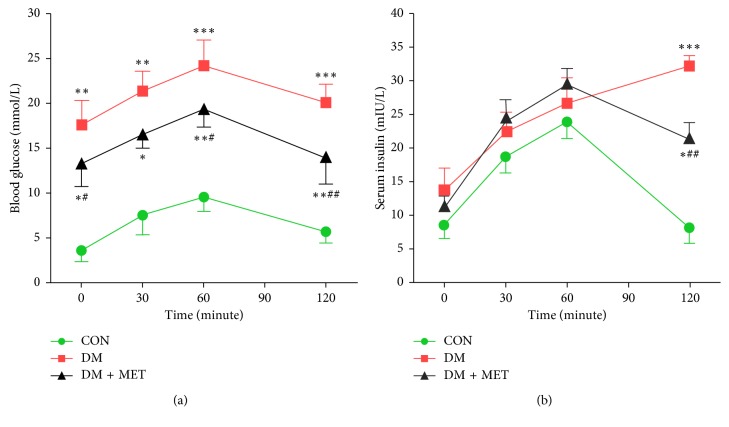
Effects of metformin on HFD/STZ-induced type 2 diabetic rats. Diabetes was induced in Wistar rats and then administered with or without metformin. At the end of the experiment, rats were fasted for 16 hours. OGTT was performed, and blood glucose (a) and serum insulin (b) at 0, 30, 60, 90, and 120 minutes were recorded. Data are mean ± SEM. ^*∗*^Mean is significantly different compared with that of the CON group. ^#^Mean is significantly different compared with that of the DM group (^*∗*^*p* < 0.05; ^*∗∗*^*p* < 0.01; ^*∗∗∗*^*p* < 0.001; ^#^*p* < 0.05; ^##^*p* < 0.01).

Based on previous studies, we established a T2DM model by feeding a HFD and intraperitoneal injection of a small dose of STZ. The levels of FBG and insulin were measured from sera collected by puncturing the retro-orbital plexus at week 24. The results demonstrated that DM rats had high levels of FBG and high fasting insulin levels compared to the CON rats (Figures 2(a) and 2(b); *p* < 0.01). Oral glucose tolerance test (OGTT) showed that glucose concentration in DM rats was higher than that in CON rats at any time point (Figures 2(a)) and insulin secretion was delayed. DM rats showed severe insulin resistance, which was consistent with the pathophysiological characteristics of type 2 diabetes. Administering metformin to DM + MET rats for 12 weeks effectively reduced FBG levels, and insulin secretion peak appeared earlier. This suggests that metformin can improve insulin resistance under the current experimental conditions.

As shown in Table 2, TC, TG, TBAs, and LDL-C were markedly increased in the DM rats, suggesting that DM rats have disordered blood lipids and bile acids. After 12 weeks of treatment with metformin, the levels of TC, TG, TBAs, and LDL-C were significantly lower than those of DM rats (*p* < 0.01). The administration of metformin results in a decrease in bile acid reabsorption. Since bile acids are converted from cholesterol in the liver, cholesterol can be excreted into the intestine through bile acid secretion and finally excreted in the feces, thereby promoting the excretion of bile acids in the body.

### 3.5. Effect of Metformin on the Expression of Genes Involved in Cholic Acid Synthesis in Diabetic Rats

**Figure 3:**
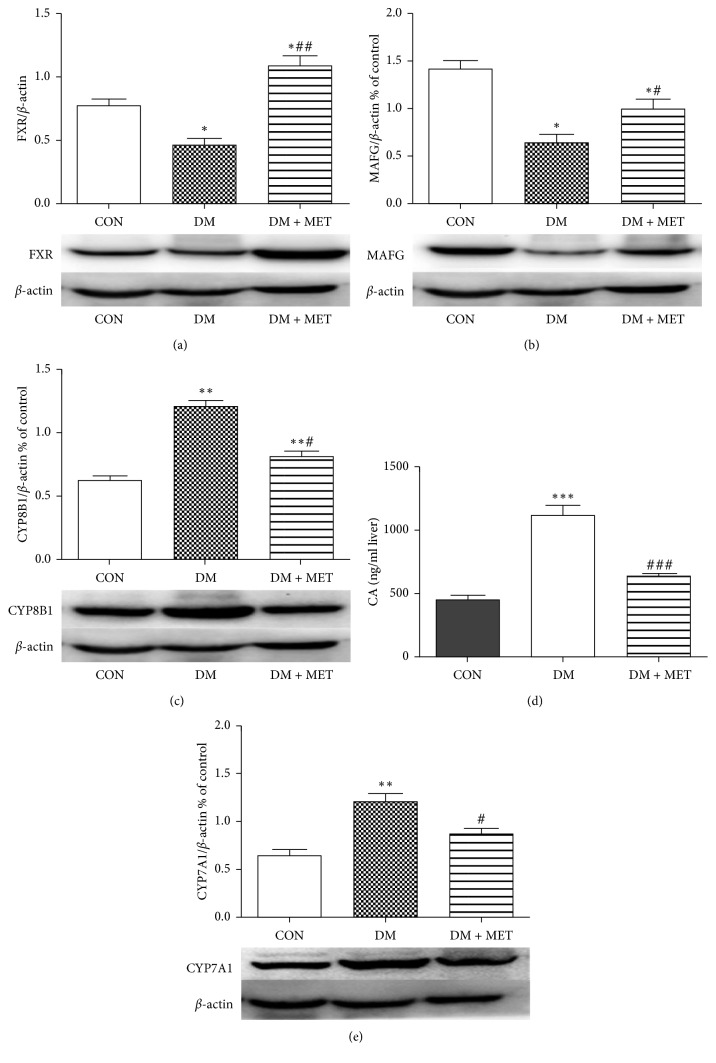
Effects of metformin on FXR, MAFG, CYP8B1, and CYP7A1 in liver tissue induced diabetes in Wistar rats. Protein contents were detected by western blotting. FXR (a), MAFG (b), CYP8B1 (c), and CYP7A1 (e) in liver tissue were quantified using Image J. The data shown represents 3–5 independent experiments. ^*∗*^Mean values were significantly different (*p* < 0.05) compared to CON rats. ^#^Mean values were significantly different (*p* < 0.05) compared to DM rats. (d) The content of cholic acid in liver tissue was determined by ELISA. Data are expressed as mean ± SEM (^*∗∗∗*^*p* < 0.001, ^###^*p* < 0.001).

It has been found that metformin affects FXR, MAFG, and CYP8B1 at the cellular level. We further investigated whether metformin affects FXR, MAFG, CYP8B1, and CYP7A1 in liver tissue levels of diabetic rats. At 32 weeks of age, FXR (Figure 3(a)) and MAFG (Figure 3(b)) levels were reduced in DM rats, while CYP8B1 (Figure 3(c)) and CYP7A1 (Figure 3(e)) were increased. In contrast, after metformin treatment, we detected an increase in FXR (Figure 3(a)) and MAFG (Figure 3(b)) and a decrease in CYP8B1 (Figure 3(c)) and CYP7A1 (Figure 3(e)) in DM + MET rats compared with DM rats.

### 3.6. Effect of Metformin on Cholic Acid Synthesis in Diabetic Rat

We quantified cholic acid in liver tissue as we observed decrease in the expression of CYP8B1 reflecting cholic acid synthesis and an increase in the expression of MAFG and FXR in CYP8B1 regulation after metformin treatment. It occurred that the concentration of cholic acid (Figure 3(d)) in DM rats was obviously more than that in CON rats, and the cholic acid (Figure 3(d)) content was lower than that of DM rats after metformin intervention.

## 4. Discussion

In the research, we established a rat model with T2DM by HFD and small-dose STZ injection. It is reported that HFD causes insulin resistance, STZ is toxic to islet *β* cells, and injection of large doses of STZ can damage most islet *β* cells to induce T1DM, while our laboratory demonstrated that low-dose STZ can be used to prepare T2DM models [14, 15].

In this study, we proved that metformin inhibits the expression of CYP8B1, thereby inhibiting the synthesis of CA, as a novo target for the treatment of type 2 diabetes. The activity of Cyp8b1 was decreased in DM rats after metformin administration, which in turn led to a decrease in the level of CA. At the same time, we observed a decrease in weight gain and an improvement in insulin resistance in diabetic rats after metformin treatment. This may be due to a correlation between 12*α*-hydroxylated BA levels (mainly CA) and insulin sensitivity [1].

A growing number of research is interested in the effects of metformin on endogenous BA, and whether BA signaling can explain its effect on improving insulin sensitivity, so we determined that metformin regulates the pathway of sterol 12*α*-hydroxylase CYP8B1. Metformin is effective in lowering blood glucose [16], reducing body weight [17], and improving insulin resistance [18] and has been widely studied for its importance in the treatment of diabetes [19]. The main pathway for cholesterol catabolism is the formation of bile acids [20]. CYP7A1 is the first rate-limiting enzyme in the classical pathway for bile acid synthesis, producing two major bile acids, cholic acid, and chenodeoxycholic acid. CYP8B1 catalyzes the synthesis of CA and plays a central role in intestinal cholesterol absorption and cholesterol gallstones, dyslipidemia, and the pathogenesis of diabetes [21]. FXR is thought to be the primary regulator of this steady-state process, inhibiting bile acid synthesis, while increasing bile acid secretion in hepatocytes and regulating bile acid content in the liver [3]. By modulating the FXR signal, bile acids themselves can act as signaling molecules that affect blood sugar and lipid metabolism [20]. In our study, FXR was activated after metformin intervention and showed a drop in blood glucose. The mechanism may be that activation of FXR increases glycogen synthesis and reduces glycolysis. In addition, FXR protects islet beta cell activity and affects glucose regulation [15]. In the liver, FXR activation induces SHP and leads to a decrease in CYP7A1 activity. Our experiments also confirmed this, activation of FXR after metformin administration resulted in decreased expression of CYP7A1 (Figure 3(e), *p* < 0.05). In the intestine, bile acid stimulates FXR to upregulate the expression of FGF19 (FGF15 in rodents) in intestinal epithelial cells [22, 23]. We observed a decrease in CYP8B1 expression after metformin administration, but the principle is still indeterminate. de Aguiar Vallim et al. [8, 9] discovered a new FXR-regulated transcriptional inhibitor, MAFG, which inhibits BA metabolism by inhibiting CYP8B1 expression. The MAFG gene is reported to be a direct target of FXR, which in turn inhibits many genes involved in bile acid synthesis and metabolism. In particular, de Aguiar Vallim et al. [8] indicated that overexpression of MAFG inhibited the activity of hepatic CYP8B1, thereby reducing the contents of CA and increasing the contents of MCA. In addition, knocking out the MAFG gene increases the contents of CYP8B1 and CA [8, 9].

We found that metformin administration may affect the pathways involving FXR, MAFG, and CYP8B1. In vitro, we detected the expression of FXR, MAFG, and CYP8B1 in HepG2 cells after metformin administration and found that metformin increased the expression of FXR and MAFG in HepG2 cells in a time- and dose-dependent manner, while decreasing the expression of CYP8B1.

When completed in vivo experiments, HFD/STZ-induced type 2 diabetic models showed elevated blood sugar and delayed peak serum insulin at all time points. Therefore, the diabetic rat model induced by HFD/STZ was in an insulin resistant state and was still able to secrete large amounts of insulin. Metformin significantly improved FBG and insulin resistance in diabetic rats. In addition, lipid mass spectrometry such as LDL-C and TG was ameliorated after metformin intervention. In vivo, we demonstrate that FXR and MAFG are reduced and CYP8B1 is elevated in HFD/STZ-treated diabetic rats (Figure 3, all *p* < 0.05), which resemble to previous research studies [7]. It has been reported that the level of Cyp8b1 mRNA and enzyme activity is increased in streptozotocin-induced diabetic rats [1]. High cholesterol diet in diabetic rats further increased cholesterol to bile acid conversion and CA/CDCA ratio [24, 25].

Our current study showed that FXR and MAFG expression was increased while CYP8B1 expression was decreased in DM + MET rats after metformin administration. Our results contradict a research which found that metformin-activated AMPK downregulates FXR transcriptional activity, thereby disrupting bile acid homeostasis and impairing the liver [26]. We hypothesize that this is because our animal model is different from the cholestasis model.

To assess the effect of metformin on bile acid metabolism, we measured serum bile acids that reflect systemic BA levels. In this work, we surveyed elevated serum BA levels in diabetic rats, suggesting a disorder of bile acid metabolism, which is consistent with our previous studies [15]. The decrease in total serum BA levels in the DM + MET group after metformin treatment was due to the decreased expression of CYP7A1, a key enzyme involved in bile acid synthesis, which inhibited the synthesis of BA. The major products of the BA synthesis pathway in humans are CDCA and CA. In rodents, most CDCA is converted to MCA [27]. CA produces secondary bile acid DCA with the effect of the intestinal microbiota. CA, DCA, TDCA, and TCA are 12*α*-hydroxylated BAs catalyzed by CYP8B1 [27]. Biddinger and Kahn [28] observed an increase in BA levels catalyzed by serum 12*α*-hydroxylated BA compared to the control group. Haeusler's research [29] showed that insulin resistance is associated with increased levels of serum 12*α*-hydroxylated BA. Kaur et al. demonstrated that loss of CA resulted in an improvement in insulin resistance in Cyp8b1^−/−^ mice [30]. In conclusion, there is a striking relativity between 12*α*-hydroxylated BA and insulin resistance. Based on CA being the most abundant 12*α*-hydroxylated BA in liver tissue, we used an ELISA kit to measure the CA content of liver tissue in a diabetic rat model. We found that the CA content of diabetic rats increased significantly, and the CA content decreased after metformin administration. These results are consistent with the expression of CYP8B1 at the genetic level. After administration of metformin, the expression of CYP8B1 is decreased and the CA content is reduced. The above findings may be important for metformin to improve insulin resistance and further explore the novel mechanism of action of metformin. Our research is based primarily on animal studies, and the clinical impact of metformin on bile acid metabolism homeostasis remains to be determined in humans.

## Figures and Tables

**Table 1:** Changes in fluid and food intake and body weight of rats in each group after STZ injection.

Groups	Fluid intake (mL/d)	Food intake (g/d)	Weight gain (g/d)
CON	22.00 ± 2.00	19.70 ± 0.40	2.47 ± 0.10
DM	112.00 ± 2.00^a^	31.60 ± 0.30^a^	1.50 ± 0.30^a^
DM + MET	32.00 ± 4.00^ab^	22.80 ± 0.10^ab^	−1.05 ± 0.50^ab^

After STZ injection, metformin decreased food intake and body weight gain in rats. Data are mean ± SEM for twenty animals in each group. ^a^*p* < 0.05 versus CON rats. ^b^*p* < 0.05 vs. DM rats. CON rats: control rats group; DM rats: HFD/STZ induced diabetic rats group; DM + METF rats: HFD/STZ-induced diabetic rats supplemented with metformin treatment.

**Table 2:** Biochemical characteristics after administration of metformin.

Parameters	CON	DM	DM + MET
Body weight (g)	482.56 ± 40.12	376.28 ± 44.87^a^	336.43 ± 38.79^ab^
FPG (mmol/L)	4.67 ± 0.86	17.49 ± 2.77^aa^	14.26 ± 5.72^ab^
FIN (m IU/L)	7.11 ± 0.79	12.58 ± 0.93^a^	10.75 ± 0.39^ab^
TG (mmol/L)	1.38 ± 0.58	3.97 ± 1.03^a^	3.79 ± 0.87^a^
TC (mmol/L)	2.07 ± 0.28	10.46 ± 3.62^aa^	3.08 ± 0.98^ab^
LDL-C (mmol/L)	0.65 ± 0.21	6.83 ± 0.24^a^	0.91 ± 0.34^ab^
HDL-C (mmol/L)	2.12 ± 0.41	1.08 ± 0.17^a^	1.46 ± 0.25^ab^
TBAs (*μ*mol/L)	30.66 ± 1.93	102.75 ± 5.18^a^	77.63 ± 3.28^ab^
HOMA-IR	1.48 ± 0.44	9.78 ± 2.27^a^	6.81 ± 2.98^ab^

The body weight (BW), fasting plasma glucose (FPG), fasting insulin (FIN), total cholesterol (TC), high-density lipoprotein-cholesterol (HDL-C), low-density lipoprotein-cholesterol (LDL-C), total bile acids (TBAs), and triglycerides (TG) levels were analyzed using cobas 8000 automatic biochemistry analyzer. HOMA-IR = [FBG(mmol/L) × FINS(*μ*U/mL)]/22.5. Data are expressed as mean ± SEM. ^a^*p* < 0.05, ^aa^*p* < 0.01 vs. CON rats. ^b^*p* < 0.05 vs. DM rats. CON group: control rats group; DM group: HFD/STZ-induced diabetic rats group; DM + METF rats: HFD/STZ-induced diabetic rats supplemented with metformin treatment.

## Data Availability

The data used to support the findings of this study are included within the article.
